# Supplementary material for: Diverse ancestral representation improves genetic intolerance metrics
Source: Nat Commun. 2025 Mar 18;16:2648. doi: 10.1038/s41467-025-57885-5 (PMC11920395; doi:10.1038/s41467-025-57885-5)
Supplement: Supplementary file 1 — Supplementary Information [file 41467_2025_57885_MOESM1_ESM.pdf]

Supplemental Information

Diverse ancestral representation improves genetic intolerance metrics

Alexander L. Han[1,2], Chloe F. Sands[1,2], Dorota Matelska[3], Jessica C. Butts[4,5], Vida Ravanmehr[1,2], Fengyuan Hu[3], Esmeralda Villavicencio Gonzalez[2,6], Nicholas Katsanis[7], Carlos D. Bustamante[7], Quanli Wang[8], Slavé Petrovski*[3,9], Dimitrios Vitsios[3], Ryan S. Dhindsa*[1,2,6]

1. Department of Pathology and Immunology, Baylor College of Medicine, Houston, Texas 77030, USA
2. Jan and Dan Duncan Neurological Research Institute, Texas Children's Hospital, Houston, TX 77030, USA
3. Centre for Genomics Research, Discovery Sciences, BioPharmaceuticals R&D, AstraZeneca, Cambridge, UK
4. Department of Bioengineering, George R. Brown School of Engineering, Rice University, Houston, TX, 77005, USA
5. Rice Neuroengineering Initiative, George R. Brown School of Engineering, Rice University, Houston, TX, 77030, USA
6. Department of Molecular and Human Genetics, Baylor College of Medicine, Houston, TX 77030, USA
7. Galatea Bio, Inc. Miami, FL 33016
8. Centre for Genomics Research, Discovery Sciences, BioPharmaceuticals R&D, AstraZeneca, Waltham, MA, US
9. Department of Medicine, Austin Health, University of Melbourne, Melbourne, Australia

These authors contributed equally: Alexander L. Han, Chloe F. Sands
Correspondence: Ryan S. Dhindsa (ryan.dhindsa@bcm.edu) and Slavé Petrovski (slav.petrovski@astrazeneca.com)

Supplementary Table 1. Abbreviation Glossary

AFR	Africa
AMR	Admixed American
SAS	South Asian
EAS	East Asian
ASJ	Ashkenazi Jewish
NFE	non-Finnish European
FIN	Finnish
LOF	Loss of Function
PTV	Protein Truncating Variant
RVIS	Residual Variance Intolerance Score
MTR	Missense Tolerance Ratio
DEE	Developmental and Epileptic Encephalopathy
DD	Developmental Delay
ASD	Autism Spectrum Disorder
NDD	Neurodevelopmental Disorder

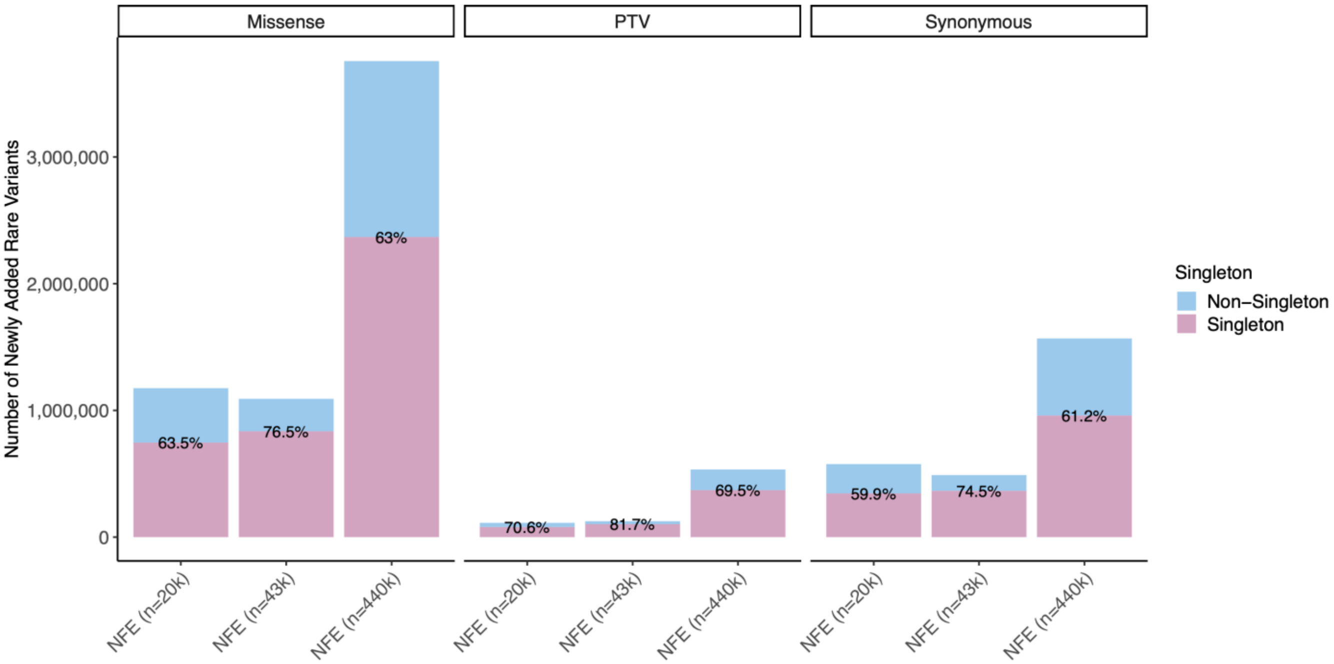

Supplementary Fig. 1. Ratio of singletons among rare variants (MAF < 0.05%) in the NFE 20k, 43k, and 440k cohorts. Figure demonstrates the number of newly detected missense, PTV, and synonymous variants with increased sample size. The singleton proportion of these newly added variants area also indicated by the color distinction and percent. NFE = non-Finnish European; PTV = Protein Truncating Variant.

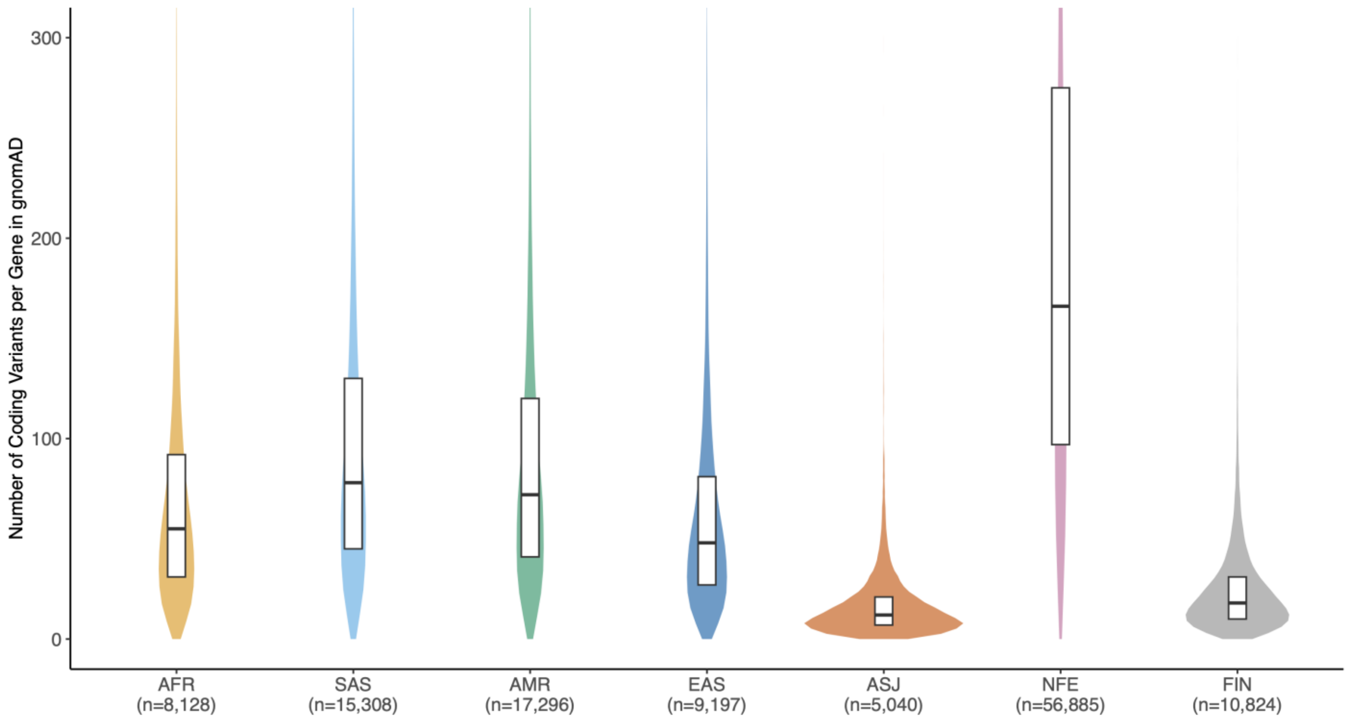

Supplementary Fig. 2. Number of coding variants detected per gene across gnomAD ancestry groups. These coding variants were not restricted to either common or rare variants and included synonymous, missense, and LOF variants. The boxplots show the median (centre line) and interquartile ranges (IQR) (box limits). AFR = African; SAS = South Asian; AMR = Admixed American; EAS = East Asian; ASJ = Ashkenazi Jewish; NFE = non-Finnish European; FIN = Finnish.

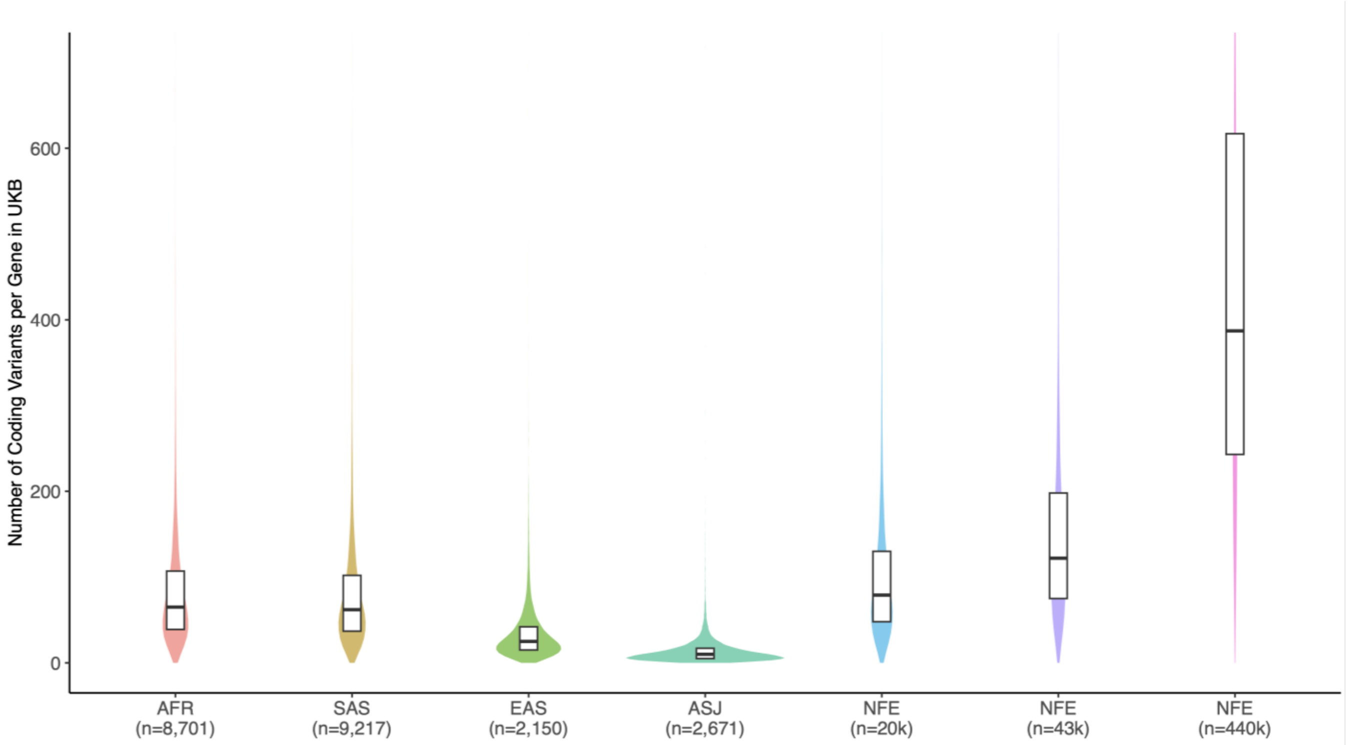

Supplementary Fig. 3. Number of coding variants detected per gene across UKB ancestry groups. These coding variants were not restricted to either common or rare variants and included synonymous, missense, and LOF variants. The boxplots show the median (centre line) and interquartile ranges (IQR) (box limits). AFR = African; ASJ = Ashkenazi Jewish; EAS = East Asian; SAS = South Asian; NFE = non-Finnish European.

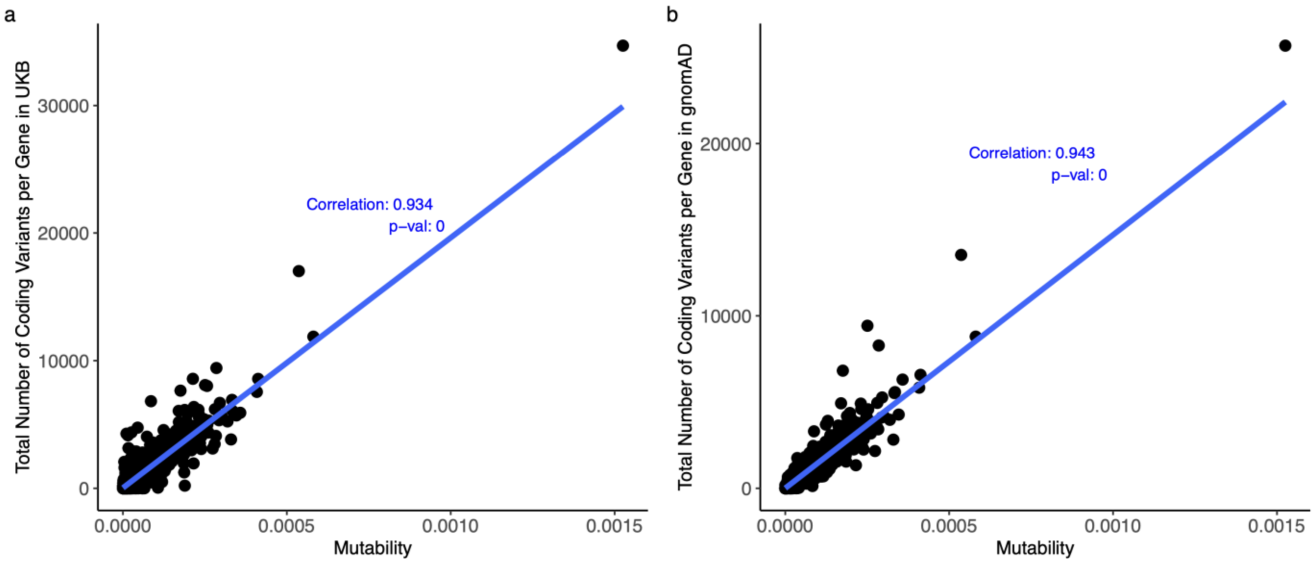

Supplementary Fig. 4. Correlation between mutability and total number of coding variants per gene for UKB [a] and gnomAD [b] cohorts. Mutability values were compared with total number of observed variants for the given gene in UKB and gnomAD cohorts. Correlation and p-value calculated via Pearson's *r*.

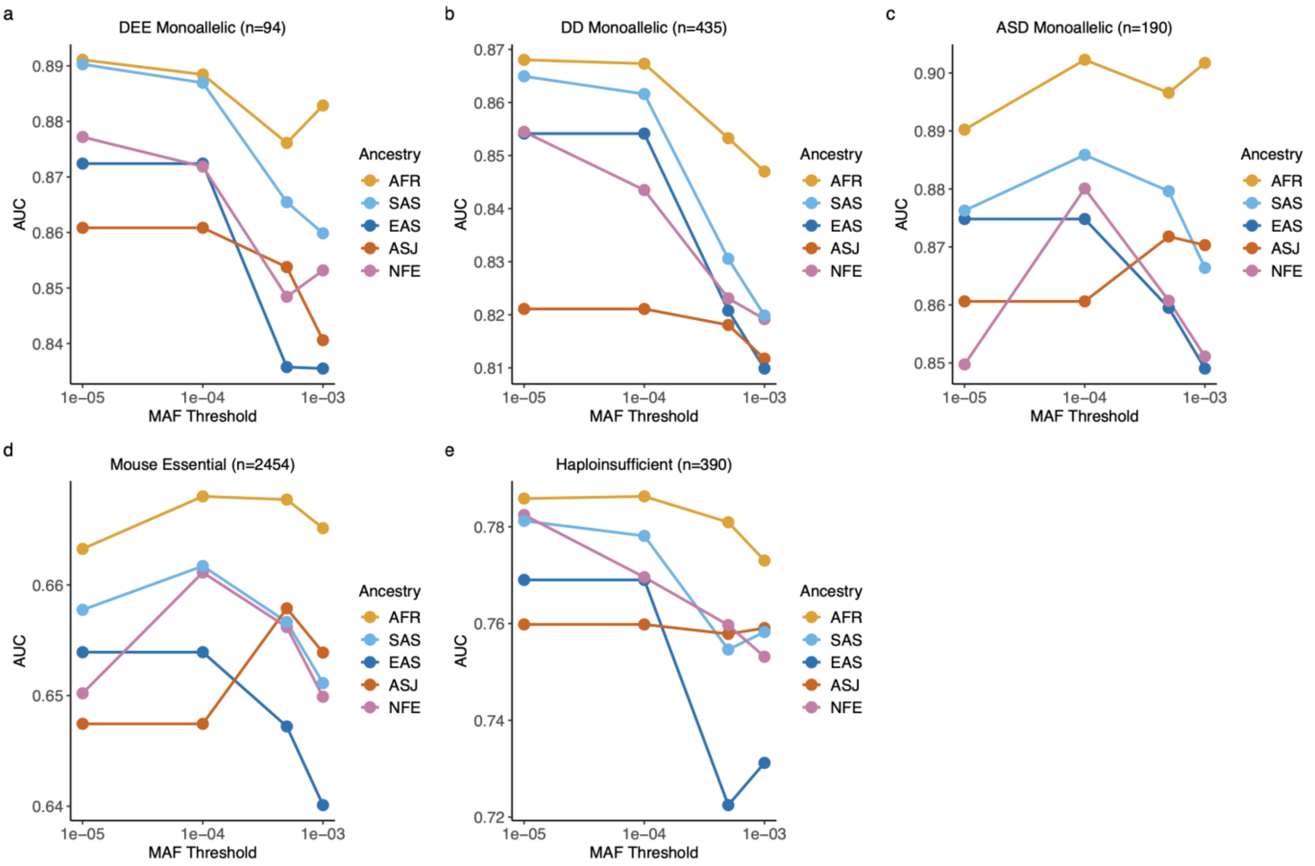

Supplementary Fig. 5 Sensitivity analysis to determine MAF cut-off. RVIS scores were computed by varying MAF cut-offs. Performance of the RVIS scores were evaluated across different gene-lists. The AUC-ROC scores were evaluated from our logistic regression models. Sensitivity analysis validated our MAF cut-off value of 0.05%. MAF = Minor allele frequency; AFR = African; ASJ = Ashkenazi Jewish; EAS = East Asian; SAS = South Asian; NFE = non-Finnish European; DEE = developmental and epileptic encephalopathy; DD = developmental delay; ASD = autism spectrum disorder.

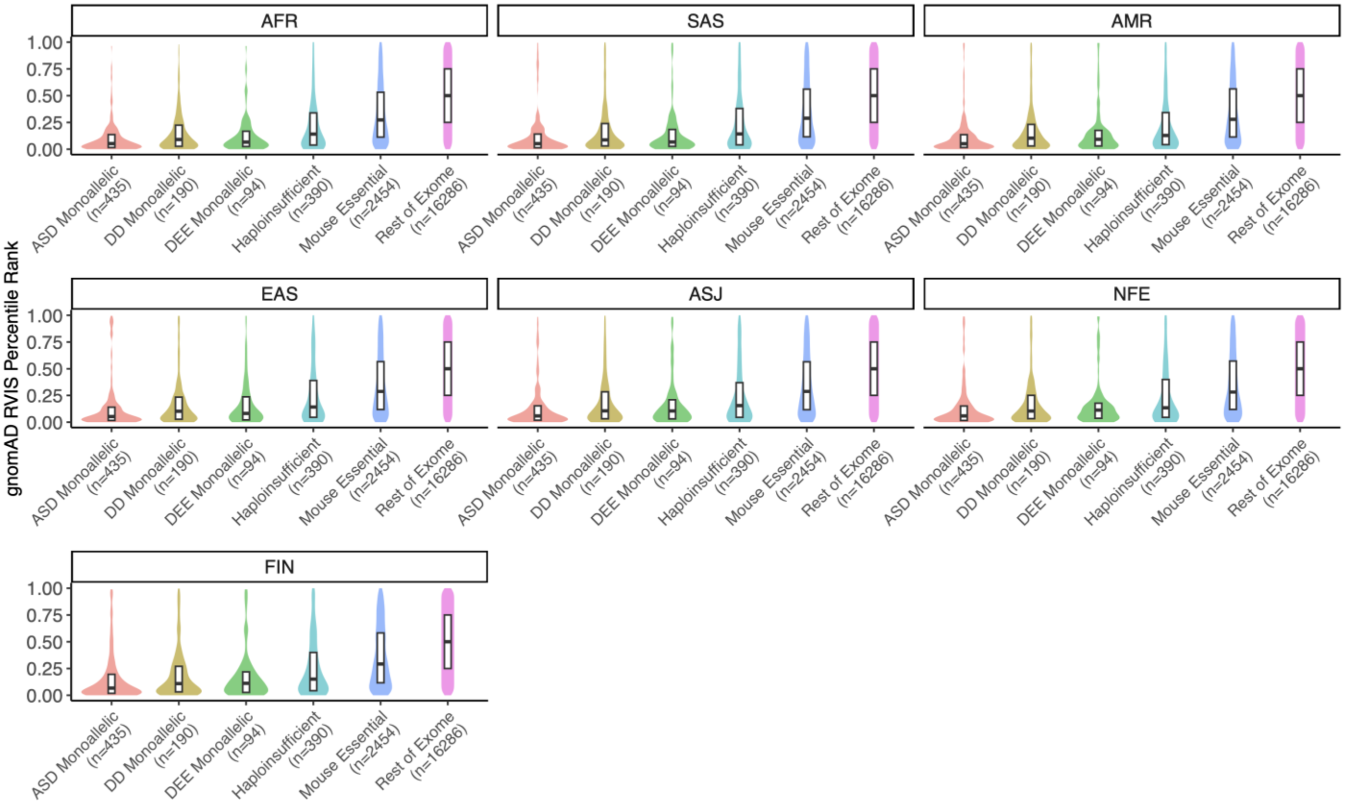

Supplementary Fig. 6. Distribution of gnomAD ancestry group-specific RVIS percentile rank for varying gene lists. Lower percentile rank indicates higher intolerance. Genes in the rest of the exome exclude genes included in ASD monoallelic, DD monoallelic, DEE monoallelic, haploinsufficient, and mouse essential gene lists. The boxplots show the median (centre line) and interquartile ranges (IQR) (box limits). AFR = African; AMR = Admixed American; ASJ = Ashkenazi Jewish; EAS = East Asian; FIN = Finnish; NFE = non-Finnish European; SAS = South Asian; DEE = developmental and epileptic encephalopathy; DD = developmental delay; ASD = autism spectrum disorder.

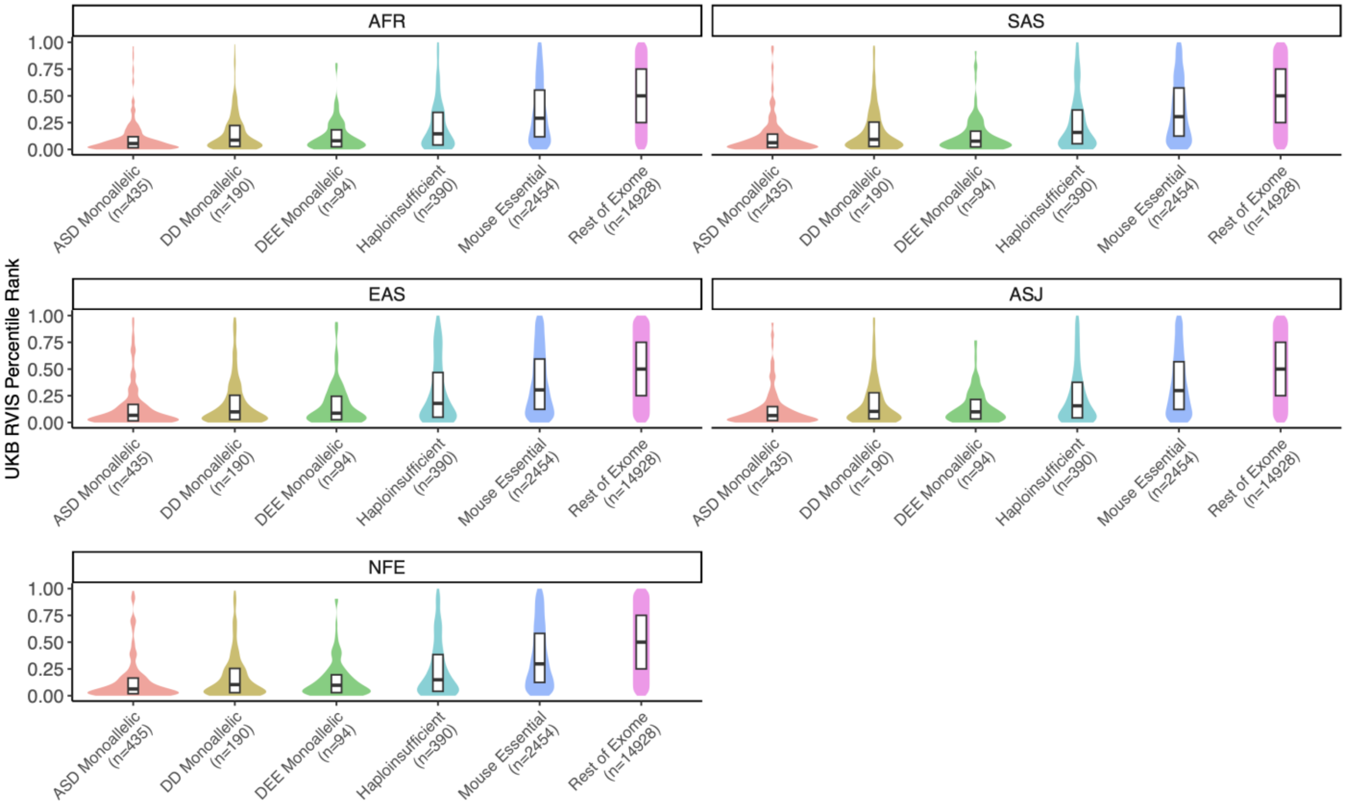

Supplementary Fig. 7. Distribution of UKB ancestry group-specific RVIS percentile rank for varying gene lists. Lower percentile rank indicates higher intolerance. Genes in the rest of the exome exclude genes included in ASD monoallelic, DD monoallelic, DEE monoallelic, haploinsufficient, and mouse essential gene lists. The boxplots show the median (centre line) and interquartile ranges (IQR) (box limits). AFR = African; ASJ = Ashkenazi Jewish; EAS = East Asian; SAS = South Asian; NFE = non-Finnish European; DEE = developmental and epileptic encephalopathy; DD = developmental delay; ASD = autism spectrum disorder.

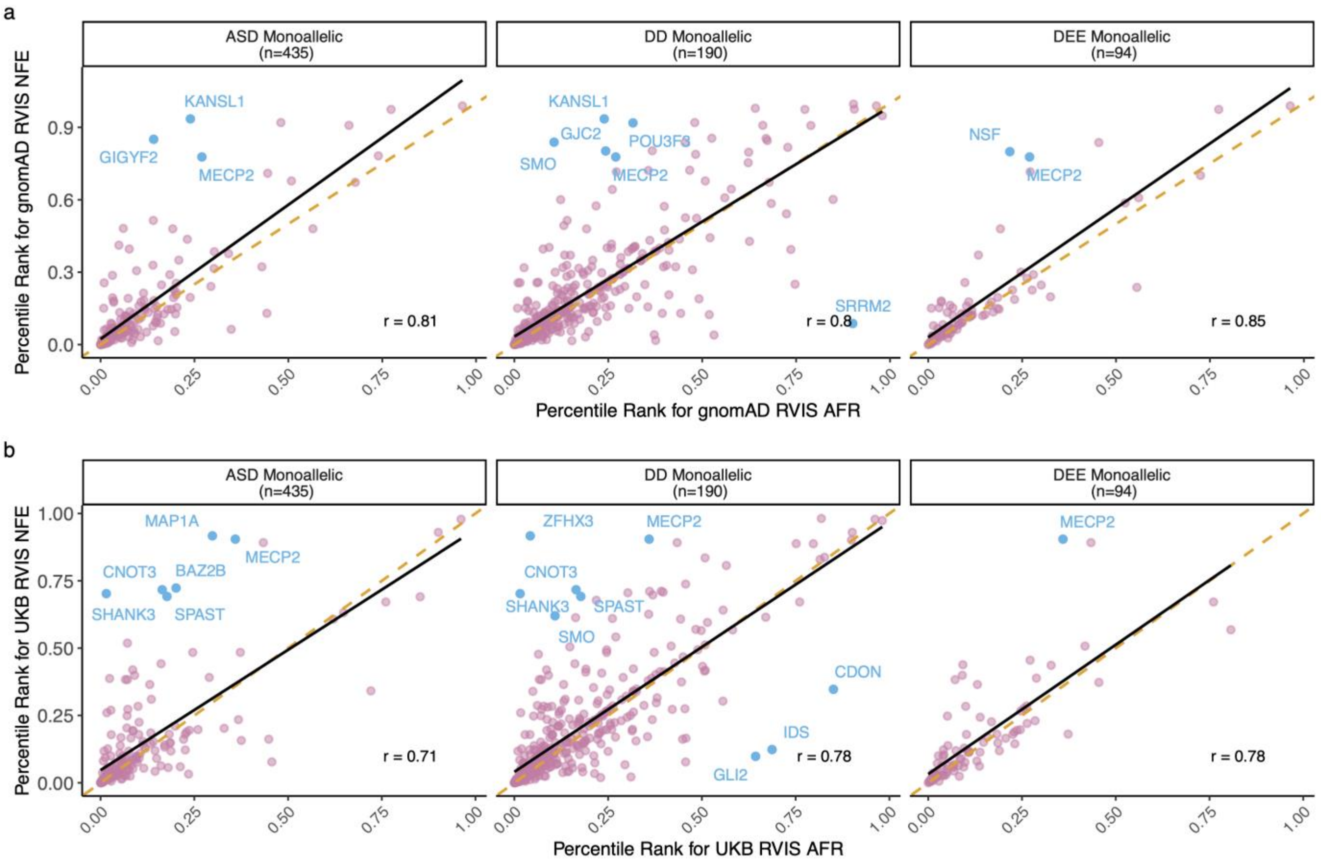

Supplementary Fig. 8. Percentile ranks for AFR- and NFE-specific RVIS scores in [a] gnomAD and [b] UKB. Lower percentile rank indicates higher intolerance. The blue point denotes genes that had percentile rank differences greater than 50%. Correlation calculated via Pearson's *r*. AFR = African; NFE = non-Finnish European; DEE = developmental and epileptic encephalopathy; DD = developmental delay; ASD = autism spectrum disorder.

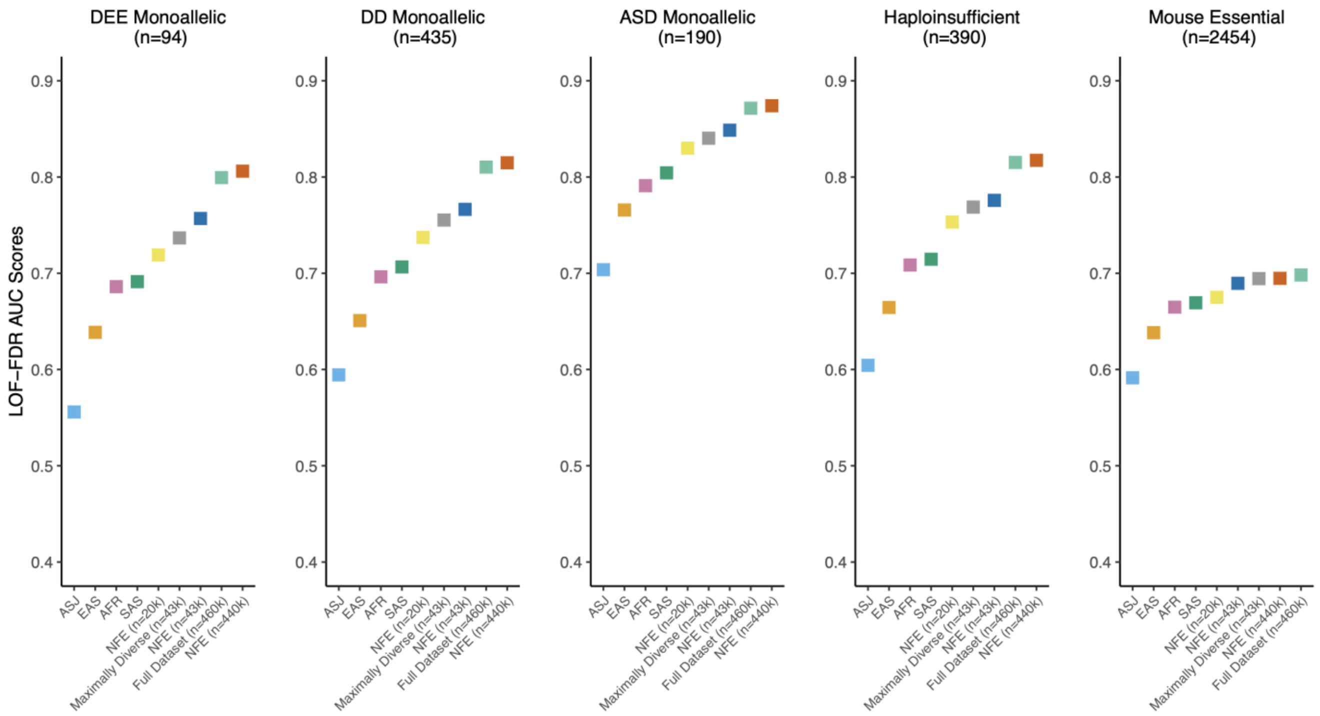

Supplementary Fig. 9. Performance of LOF-FDR trained on different UKB cohorts. AUC-ROC scores illustrate the ability of LOF-FDR to predict five different gene lists. Each score represents a version of the score trained on the UKB cohorts composed of different ancestries. AFR = African; ASJ = Ashkenazi Jewish; EAS = East Asian; SAS = South Asian; NFE = non-Finnish European; DEE = developmental and epileptic encephalopathy; DD = developmental delay; ASD = autism spectrum disorder.
