# Supplementary material for: Diverse ancestral representation improves genetic intolerance metrics
Source: Nat Commun. 2025 Mar 18;16:2648. doi: 10.1038/s41467-025-57885-5 (PMC11920395; doi:10.1038/s41467-025-57885-5)
Supplement: Supplementary file 2 — Description of Additional Supplementary Files [file 41467_2025_57885_MOESM2_ESM.pdf]

Description of Additional Supplementary Files

Supplementary Data 1 – UKB RVIS Scores

Provided as external file. Genes and UKB-derived RVIS scores are described in the table. Variable “x” accounts for total number of variants. Variable “y” indicates respective counts by ancestry (AFR = African, ASJ = Ashkenazi Jewish, EAS = East Asian, SAS = South Asian, NFE = non-Finnish European). Mutability values for each gene was derived from the gnomAD metrics table.

Supplementary Data 2 – gnomAD RVIS Scores

Provided as external file. Genes and gnomAD-derived RVIS scores are described in the table. Variable “x” accounts for total number of variants. Variable “y” indicates respective counts by ancestry (AFR = African, AMR = Admixed American, ASJ = Ashkenazi Jewish, EAS = East Asian, FIN = Finnish, NFE = non-Finnish European, SAS = South Asian). Mutability values for each gene was derived from the gnomAD metrics table.

Supplementary Data 3 – Gene Lists

Provided as external files. Each gene-list is present on separate sheets. From left to right: DD Monoallelic, DEE Monoallelic, ASD Monoallelic, Clingen Haploinsufficiency, and Mouse Essential.

Supplementary Data 4 – Logistic regression & DeLong test for UKB-derived RVIS

Provided as external files. In the Logistic Regression sheet, ancestry group-specific RVIS scores, gene-list, p-value, and corresponding AUC values provided (AFR = African, ASJ = Ashkenazi Jewish, EAS = East Asian, SAS = South Asian, NFE = non-Finnish European). In the DeLong Test sheet, gene-list, score1, score2, p-value, and adjusted p-value are provided**.** P-values were generated via a two-sided DeLong test. The adjusted p-value column represents Benjamini-Hochberg corrected p-values.

Supplementary Data 5 – Logistic regression & DeLong test for gnomAD-derived RVIS

Provided as external files. In the Logistic Regression sheet, ancestry group-specific RVIS scores, gene-list, and corresponding AUC values provided (AFR = African, AMR = Admixed American, ASJ = Ashkenazi Jewish, EAS = East Asian, FIN = Finnish, NFE = non-Finnish European, SAS = South Asian). In the DeLong Test sheet, gene-list, score1, score2, p-value, and adjusted p-value are provided. P-values were generated via a two-sided DeLong test. The adjusted p-value column represents Benjamini-Hochberg corrected p-values.

Supplementary Data 6 – Percentile Rank Scores of gnomAD- and UKB-derived RVIS

Provided as external file. In the gnomAD sheet, genes and gnomAD-derived RVIS percentile ranks are described in the table (AFR = African, AMR = Admixed American, ASJ = Ashkenazi Jewish, EAS = East Asian, FIN = Finnish, NFE = non-Finnish European, SAS = South Asian). In the UKB sheet, genes and UKB-derived RVIS percentile ranks are described in the table (AFR = African, ASJ = Ashkenazi Jewish, EAS = East Asian, SAS = South Asian, NFE = non-Finnish European).

Supplementary Data 7 – UKB MTR Scores

Provided as external file. Genes and UKB-derived MTR scores are described in the table. Variables “mis” and “syn” indicate counts of missense and synonymous variants for the individual ancestry and compiled groups (AFR = African, ASJ = Ashkenazi Jewish, EAS = East Asian, SAS = South Asian, NFE = non-Finnish European, Maximally Diverse n=43k, NFE only n=20k, NFE only n=43k, NFE only n=440k, and Full Dataset

n=460k). Variables “possible_mis” and possible_syn” account for expected counts of missense and synonymous variants.

Supplementary Data 8 – Logistic regression & DeLong test for UKB-derived MTR

Provided as external files. In the Logistic Regression sheet, cohort-specific MTR scores, gene-list, p-value, and corresponding AUC values provided (AFR = African, ASJ = Ashkenazi Jewish, EAS = East Asian, SAS = South Asian, NFE = non-Finnish European). In the DeLong Test sheet, gene-list, score1, score2, p-value, and adjusted p-value are provided. P-values were generated via a two-sided DeLong test. The adjusted p-value column represents Benjamini-Hochberg corrected p-values.

Supplementary Data 9 – UKB LOF O/E

Provided as external file. Genes and UKB-derived LOF O/E scores are described in the table. Variable “lof” indicate respective counts of LOF variants for the individual ancestry and compiled groups (AFR = African, ASJ = Ashkenazi Jewish, EAS = East Asian, SAS = South Asian, NFE = non-Finnish European, Maximally Diverse n=43k, NFE only n=20k, NFE only n=43k, NFE only n=440k, and Full Dataset n=460k). Variable “total” indicate respective counts of total variants for the individual ancestry and compiled groups. Variable “possible_lof” accounts for expected counts of LOF variants. Variable “mu_lof” was derived from the gnomAD metrics table and accounts for mutability values for LOF variants in a gene.

Supplementary Data 10 – UKB LOF-FDR

Provided as external file. Genes and UKB-derived LOF-FDR scores are described in the table. Variable “lof” indicate respective counts of LOF variants for the individual ancestry and compiled groups (AFR = African, ASJ = Ashkenazi Jewish, EAS = East Asian, SAS = South Asian, NFE = non-Finnish European, Maximally Diverse n=43k, NFE only n=20k, NFE only n=43k, NFE only n=440k, and Full Dataset n=460k). Variable “total” indicate respective counts of total variants for the individual ancestry and compiled groups. Variable “possible_lof” accounts for expected counts of LOF variants. Variable “exp_lof_percent” was calculated using mutability values from the gnomAD metrics table (Methods).

Supplementary Data 11 – Logistic regression & DeLong test for UKB-derived LOF O/E

Provided as external files. In the Logistic Regression sheet, cohort-specific LOF O/E scores, gene-list, p-value, and corresponding AUC values provided (AFR = African, ASJ = Ashkenazi Jewish, EAS = East Asian, SAS = South Asian, NFE = non-Finnish European). In the DeLong Test sheet, gene-list, score1, score2, p-value, and adjusted p-value are provided. P-values were generated via a two-sided DeLong test. The adjusted p-value column represents Benjamini-Hochberg corrected p-values.

Supplementary Data 12 – Logistic regression & DeLong test for UKB-derived LOF-FDR

Provided as external files. In the Logistic Regression sheet, cohort-specific LOF-FDR scores, gene-list, p-value, and corresponding AUC values provided (AFR = African, ASJ = Ashkenazi Jewish, EAS = East Asian, SAS = South Asian, NFE = non-Finnish European). In the DeLong Test sheet, gene-list, score1, score2, p-value, and adjusted p-value are provided. P-values were generated via a two-sided DeLong test. The adjusted p-value column represents Benjamini-Hochberg corrected p-values.
