# Supplementary material for: Diverse ancestral representation improves genetic intolerance metrics
Source: Nat Commun. 2025 Mar 18;16:2648. doi: 10.1038/s41467-025-57885-5 (PMC11920395; doi:10.1038/s41467-025-57885-5)
Supplement: Supplementary file 15 — Reporting Summary [file 41467_2025_57885_MOESM15_ESM.pdf]

Corresponding author(s): Slave Petrovski, Ryan Dhindsa

Last updated by author(s): 20/2/2025

# Reporting Summary

Nature Portfolio wishes to improve the reproducibility of the work that we publish. This form provides structure for consistency and transparency in reporting. For further information on Nature Portfolio policies, see our Editorial Policies and the Editorial Policy Checklist.

## Statistics

For all statistical analyses, confirm that the following items are present in the figure legend, table legend, main text, or Methods section.

| n/a | Confirmed | |
|---|---|---|
| ☐ | ☒ | The exact sample size (*n*) for each experimental group/condition, given as a discrete number and unit of measurement |
| ☒ | ☐ | A statement on whether measurements were taken from distinct samples or whether the same sample was measured repeatedly |
| ☐ | ☒ | The statistical test(s) used AND whether they are one- or two-sided<br>*Only common tests should be described solely by name; describe more complex techniques in the Methods section.* |
| ☒ | ☐ | A description of all covariates tested |
| ☒ | ☐ | A description of any assumptions or corrections, such as tests of normality and adjustment for multiple comparisons |
| ☐ | ☒ | A full description of the statistical parameters including central tendency (e.g. means) or other basic estimates (e.g. regression coefficient) AND variation (e.g. standard deviation) or associated estimates of uncertainty (e.g. confidence intervals) |
| ☐ | ☒ | For null hypothesis testing, the test statistic (e.g. *F*, *t*, *r*) with confidence intervals, effect sizes, degrees of freedom and *P* value noted<br>*Give P values as exact values whenever suitable.* |
| ☒ | ☐ | For Bayesian analysis, information on the choice of priors and Markov chain Monte Carlo settings |
| ☒ | ☐ | For hierarchical and complex designs, identification of the appropriate level for tests and full reporting of outcomes |
| ☐ | ☒ | Estimates of effect sizes (e.g. Cohen's *d*, Pearson's *r*), indicating how they were calculated |

*Our web collection on statistics for biologists contains articles on many of the points above.*

## Software and code

Policy information about availability of computer code

| | |
|---|---|
| Data collection | No software was used for the collection of data, as these data were already publicly available |

| | |
|---|---|
| Data analysis | Custom code for generating gene-level scores and figures is available at: https://github.com/alhanster/ancestral_diversity.<br><br>The code for generating sliding-window MTR scores and related figures is available at: https://github.com/astrazeneca-cgr-publications/OncMTR/tree/mtr-ancestry-specific. Code is also available via Zenodo (https://zenodo.org/records/14901599).<br><br>We also used the following publicly available software:<br>R 4.3.1<br>Tidyverse 2.0.0<br>data.table 1.15.4<br>R.utils 2.12.3<br>pROC 1.18.5<br>SnpEff 4.3<br>python 3.10<br>matplotlib 3.8.3<br>pandas 2.2.1<br>numpy 1.26.4 |

For manuscripts utilizing custom algorithms or software that are central to the research but not yet described in published literature, software must be made available to editors and reviewers. We strongly encourage code deposition in a community repository (e.g. GitHub). See the Nature Portfolio guidelines for submitting code & software for further information.

## Data

Policy information about availability of data

All manuscripts must include a data availability statement. This statement should provide the following information, where applicable:

- Accession codes, unique identifiers, or web links for publicly available datasets
- A description of any restrictions on data availability
- For clinical datasets or third party data, please ensure that the statement adheres to our policy

The Gene-level intolerance scores generated in this study are included in supplementary data, and the sliding window MTR scores are publicly available on FigShare (https://doi.org/10.6084/m9.figshare.26049661.v1). Scores are also browsable via our publicly available portal: http://intolerance.public.cgr.astrazeneca.com/. The UK Biobank whole-exome sequencing data are publicly available to registered researchers through the UKB data access protocol. Exomes can be found in the UKB showcase portal: https://biobank.ndph.ox.ac.uk/showcase/label.cgi?id=170. Additional information about registration for access to the data is available at http://www.ukbiobank.ac.uk/register-apply/. Data for this study were obtained under Resource Application Number 26041 and 65851. GnomAD v2.1.1 data are publicly available through the gnomAD website (https://gnomad.broadinstitute.org/data#v2). Gene lists are available through GitHub (https://github.com/alhanster/ancestral_diversity/tree/main/data/genelist); denovo-db v1.6.1 data is publicly available through https://denovo-db.gs.washington.edu/denovo-db/; and TOPMed freeze 5 data are publicly available through https://bravo.sph.umich.edu.

## Research involving human participants, their data, or biological material

Policy information about studies with human participants or human data. See also policy information about sex, gender (identity/presentation), and sexual orientation and race, ethnicity and racism.

| | |
|---|---|
| Reporting on sex and gender | All analyses included males and females. |
| Reporting on race, ethnicity, or other socially relevant groupings | We report analyses stratified by genetic ancestry group labels that were previously published. Genetic ancestry group is distinct from race and ethnicity as explained by the National Academies of Sciences, Engineering, and Medicine (NASEM). GnomAD ancestry group labels were described by Karczewski et al. (Nature, 2020). UKB ancestry group labels were described by Wang et al. (Nature, 2021). |
| Population characteristics | See above. |
| Recruitment | N/A - these data were already publicly available. |
| Ethics oversight | The protocols for UK Biobank are overseen by The UK Biobank Ethics Advisory Committee (EAC), for more information see https://www.ukbiobank.ac.uk/ethics/ and https://www.ukbiobank.ac.uk/wp-content/up1oads/2011/05/EGF20082.pdf<br><br>The gnomAD study was was overseen by the Broad Institute's Office of Research Subject Protection and the Partners Human Research Committee, and was given a determination of Not Human Subjects Research. |

Note that full information on the approval of the study protocol must also be provided in the manuscript.

# Field-specific reporting

Please select the one below that is the best fit for your research. If you are not sure, read the appropriate sections before making your selection.

☒ Life sciences ☐ Behavioural & social sciences ☐ Ecological, evolutionary & environmental sciences

For a reference copy of the document with all sections, see nature.com/documents/nr-reporting-summary-flat.pdf

# Life sciences study design

All studies must disclose on these points even when the disclosure is negative.

| | |
|---|---|
| Sample size | This study only used published datasets from prior studies. No sample size calculation was involved. The maximum number of samples available in these datasets were used whenever possible. |
| Data exclusions | None |
| Replication | We compared results from gnomAD and UKB whenever possible. We were unable to use the gnomAD dataset for the analyses that required downsampling of individual-level data because gnomAD only provides aggregate-level data. |
| Randomization | This study is observational. Blinding was not applicable to this study. |
| Blinding | This study is observational. Blinding was not applicable to this study. |

# Reporting for specific materials, systems and methods

We require information from authors about some types of materials, experimental systems and methods used in many studies. Here, indicate whether each material, system or method listed is relevant to your study. If you are not sure if a list item applies to your research, read the appropriate section before selecting a response.

## Materials & experimental systems

| n/a | Involved in the study |
|---|---|
| ☒ | ☐ Antibodies |
| ☒ | ☐ Eukaryotic cell lines |
| ☒ | ☐ Palaeontology and archaeology |
| ☒ | ☐ Animals and other organisms |
| ☒ | ☐ Clinical data |
| ☒ | ☐ Dual use research of concern |
| ☒ | ☐ Plants |

## Methods

| n/a | Involved in the study |
|---|---|
| ☒ | ☐ ChIP-seq |
| ☒ | ☐ Flow cytometry |
| ☒ | ☐ MRI-based neuroimaging |

## Plants

| | |
|---|---|
| Seed stocks | *Report on the source of all seed stocks or other plant material used. If applicable, state the seed stock centre and catalogue number. If plant specimens were collected from the field, describe the collection location, date and sampling procedures.* |
| Novel plant genotypes | *Describe the methods by which all novel plant genotypes were produced. This includes those generated by transgenic approaches, gene editing, chemical/radiation-based mutagenesis and hybridization. For transgenic lines, describe the transformation method, the number of independent lines analyzed and the generation upon which experiments were performed. For gene-edited lines, describe the editor used, the endogenous sequence targeted for editing, the targeting guide RNA sequence (if applicable) and how the editor was applied.* |
| Authentication | *Describe any authentication procedures for each seed stock used or novel genotype generated. Describe any experiments used to assess the effect of a mutation and, where applicable, how potential secondary effects (e.g. second site T-DNA insertions, mosiacism, off-target gene editing) were examined.* |
